# Salivary Extracellular Vesicle-Associated exRNA as Cancer Biomarker

**DOI:** 10.3390/cancers11070891

**Published:** 2019-06-26

**Authors:** Giulia Chiabotto, Chiara Gai, Maria Chiara Deregibus, Giovanni Camussi

**Affiliations:** 1 Department of Medical Sciences, University of Torino, Torino 10126, Italy; 2 2i3T Business Incubator and Technology Transfer, University of Torino, Torino 10126, Italy

**Keywords:** miRNA, non coding RNA, exosomes, microvesicles, cancer, saliva

## Abstract

Extracellular vesicles (EVs) secreted in biological fluids contain several transcripts of the cell of origin, which may modify the functions and phenotype of proximal and distant cells. Cancer-derived EVs may promote a favorable microenvironment for cancer growth and invasion by acting on stroma and endothelial cells and may favor metastasis formation. The transcripts contained in cancer EVs may be exploited as biomarkers. Protein and extracellular RNA (exRNA) profiling in patient bio-fluids, such as blood and urine, was performed to identify molecular features with potential diagnostic and prognostic values. EVs are concentrated in saliva, and salivary EVs are particularly enriched in exRNAs. Several studies were focused on salivary EVs for the detection of biomarkers either of non-oral or oral cancers. The present paper provides an overview of the available studies on the diagnostic potential of exRNA profiling in salivary EVs.

## 1. Introduction

The aim of liquid biopsy is to identify biomarkers with diagnostic, predictive and prognostic values in bio-fluids, to avoid more invasive approaches. Researchers focused on different types of biomarkers, including proteins, circulating DNA fragments and cells, and extracellular RNAs (exRNAs). ExRNAs are more sensitive and specific biomarkers than proteins and better reflect the cell dynamic than DNA does [1]. However, several limitations in the use of exRNA as biomarkers still remain, related to their heterogeneity, the incomplete definition of their multiple targets and functions, and their stability in different biological fluids [2].

Nowadays, the recently developed techniques of sequencing allow for an accurate evaluation of RNA expression, which reflects cellular genetic and functional states. Different types of RNA biomarkers have been considered in cancer. Differential mRNA expression profiles may reflect the positive and negative regulation of tumor-associated genes in several cancers and may provide suitable biomarkers for monitoring the clinical outcome of patients [3,4,5]. Non-coding RNAs, such as microRNAs (miRNAs), piwi-interacting RNA (piRNA), small nucleolar RNA (snoRNA), circular RNA (circRNA) and long non-coding RNAs (lncRNAs), have also been investigated as potential biomarkers in cancer [1]. Moreover, the detection of chimeric RNAs may allow for the identification of chromosomal aberrations [6,7]. The stability of different exRNAs in the biological fluids depends on protection from exonucleases, provided either by RNA binding proteins, such as those of the Argonaute family, and high- and low-density lipoproteins, and by encapsulation in membrane vesicles [8,9,10].

Membrane vesicles released by cells in the extracellular space have recently emerged as a good evolutionarily preserved mechanism of inter-cellular communication. The vesicles are able to share genetic information among cells by delivering proteins, bio-active lipids and nucleic acids protected from degrading enzymes [11,12,13]. These vesicles, termed extracellular vesicles (EVs), are abundant in all biological fluids and can be exploited for searching biomarkers since they retain the molecular signature of the cell of origin.

One challenge of liquid biopsy is the choice of the bio-fluid that better reflects the occurrence of cancer. Most studies have focused on blood, but several other bio-fluids are now gaining attention, including saliva. Saliva is enriched in EVs and may represent a bio-fluid suitable for searching for markers of oral and systemic diseases.

## 2. EVs as Carriers of exRNA

EVs are a heterogeneous population, which includes membrane vesicles of different sizes and biogenesis. The three main categories of EVs include exosomes, ectosomes, and apoptotic bodies [14,15,16,17]. Exosomes are nano-sized vesicles (35–100 nm), which originate from the multivesicular bodies and are secreted by a process of exocytosis. This process requires the inward budding of multivesicular bodies-membrane, followed by fusion with plasma membrane and release in the extracellular space. The endosomal sorting complex required for transport (ESCRT) machinery, and several components of the Ras GTPases (RAB) family [18,19] and of the tetraspanin family [18], are involved in such processes. Vesicles generated by the budding of surface plasma membrane with the inclusion of cytoplasmic constituents have been termed microvesicles. This term is misleading as these vesicles include a large population of vesicles within the nano-range (60–250 nm), such as those released from healthy cells. It has been therefore suggested that one should name these vesicles ectosomes or shedding vesicles [14]. Shedding vesicles also include larger vesicles that may reach 1000 nm, and some of them may derive from cells in a pre-apoptotic phase. Microvesicle formation is related to the modification of plasma membrane curvature due to changes in lipid and protein interactions involving the arrestin domain-containing protein-1 (ARRDC1) and the late endosomal protein tumor susceptibility gene 101 (*TSG101*). The cytoskeleton rearrangements controlled by the signaling cascade of Ras-related GTPase ADP-ribosylation factor 6 (ARF6) promote vesiculation and release [20]. The apoptotic bodies released by cells undergoing programmed death are vesicles with a diameter of 1000–5000 nm and may contain nuclear fragments and intact chromosomes [21].

Most of the studies on the use of EVs as potential biomarkers have been performed on exosomes and microvesicles, as both types of vesicles may encapsulate fragments of genomic and mitochondrial DNA origin [22,23,24,25], and different classes of RNA, such as mRNA, miRNA, lncRNA, mitochondrial RNA, transfer RNA, and ribosomal RNA [26,27,28,29]. Furthermore, nano-sized vesicles may be released by the same cell by exocytosis or by surface membrane budding, and it may be difficult to discriminate vesicles discharged by non-apoptotic cells on the basis of mechanisms of origin. In fact, some molecules constituent of the endosomal sorting complex required for transport (ESCRT) and some ancillary proteins such as *TSG101*, Alix and Vacuolar protein sorting-associated protein 4 (VPS4) implicated in the formation of exosomes, are also reported in the literature to also be shared by shedding vesicles [30]. The discharge of exosomes may involve some constituents of the RAB family of GTPase proteins implicated in the MVBs/plasma membrane interaction [31,32]. Furthermore, the biogenesis of shedding vesicles may depend on a reorganization of the proteins of the cytoskeleton myosin and actin under the control of the ARF6 signaling [30]. Some tetraspanins and some ESCRT proteins are often reported as common exosome and shedding vesicle markers and cannot represent a peculiarity principle [33]. However, CD9, CD63 and CD81 tetraspanins are reported to be enhanced in exosomes [18], while annexin A1 is considered a marker for microvesicles [34]. Due to the heterogeneity of EVs produced by different cell types and present in the biological fluids, the protocols used for EV purification assume a critical relevance. For this reason, the public available databases [35,36,37] take into account the procedures used for the purification of EVs when describing the lipid, protein and nucleic acid composition. Of interest, the comparative lipidomic, proteomic and genomic analyses between the cells of origin and their released EVs highlight the presence of qualitative and quantitative differences, in both basal and stimulated condition. These data suggest that the EV cargo is actively modulated [38,39,40]. The EV mediated transfer of their cargo into recipient cells can induce epigenetic and functional changes into the recipient cells [41]. Some studies indicate that genetic materials encapsulated in EV include mitochondrial [24,42] and genomic [22] DNAs. Other studies performed on exosomal sub-fractions of EVs suggest that the DNA release is not related to the small vesicle release but to the autophagy- and multivesicular-endosome-dependent mechanism [34]. Several RNA species were found to be associated with EVs. EVs contain intact mRNA that can be translated into proteins in the recipient cells [27,28], but also many fragments of 200 nucleotides [43] that may have a biological role as scavengers and/or values as biomarkers. The exRNA enriched in EVs include miRNAs, ribosomal RNAs, tRNA fragments piRNA, snoRNA, Y-RNA, circRNA and lncRNAs [44,45,46,47,48].

Little is known about the process of nucleic acid compartimentalization into EVs [39,49,50,51,52]. Some proteins involved in EV biogenesis are potential candidates for RNA encapsulation in EVs. For instance, it has been shown that in EVs purified by differential ultracentrifugation from liver stem cells, Alix coprecipitate with Argonate 2 (Ago2) protein and miRNAs. The significant reduction of EV-associated miRNAs in Alix knock-down cells suggests that Alix can have a role as a component of ESCRT in the export of the Ago2-miRNA complex [53]. Through a high-resolution density gradient fractionation coupled with an immunoaffinity capture of exosomes, Argonaute proteins were detected in the non-vesicular compartment [34], which may contain components of the multivesicular body membranes. In breast cancer-derived EVs, miRNAs associated with Ago2 were shown to induce an alteration in the transcriptome of the recipient cells [54]. By regulating the Ago2 secretion [55], GTPase KRas (KRAS) has been involved in the miRNA compartmentalization into EVs released by colorectal cancer cells [56]. Moreover, miRNA packing into EVs depends on the interaction with the heterogeneous nuclear ribonucleoprotein A2B1 (hnRNPA2B1) [57] and with the RNA-binding protein Y-box protein I (YBX1) [58].

## 3. EVs in Cancer Biology

EVs released by cancer cells may act both locally, contributing to create a favorable microenvironment for cancer growth, and at distance, promoting the metastatic niche formation. Several studies have shown that cancer EVs contribute to the induction of chemo-resistance [59,60,61,62,63], to the remodeling of extracellular matrix [64,65], to tumor vascularization [66] and to epithelial-mesenchymal transition with a consequent enhanced migration/invasion and metastasis formation [67,68,69]. EVs also participate as active players in the bi-directional crosstalk between cancer cells and cells present in the microenvironment, such as fibroblasts [70,71], which may secrete EVs conferring chemo-resistance [72,73,74] and invasiveness to cancer cells [70,71].

Several mechanisms of action involving the EV-mediated transfer of proteins and exRNA have been described and exploited as diagnostic markers. In particular, the miRNA-mediated effects have been extensively studied. Several miRNAs present in EVs released from breast cancer (miR-100, miR-222, miR30a and miR-17), lung cancer (miR-100-5p), and ovarian cancer (miR-21) or released from stromal cells (miR-21 and miR-146a) were shown to confer chemo-resistance [60,61,62,73,74] (Table 1). Cancer EVs may contribute to new blood vessel formation by transferring to recipient fibroblasts and endothelial cells pro-angiogenic miRNAs such as miR-155, miR-210 and miR-494, which are under the regulation of the hypoxia-inducible factor (HIF) 1α [75,76,77,78,79] (Table 2). Moreover, several studies on EVs released by cancer cells indicate that they promote the development of a pre-metastatic niche by transferring either proteins or oncogenic miRNAs [80,81,82,83]. For instance, some miRNAs (miR-125b, miR-130b and miR-155) present in prostate cancer and released by EVs have been shown to confer a protumorigenic phenotype to adipose-derived mesenchymal stem cells [84].

A contribution in favoring the tumor immune-escape of EVs released by cancer cells has been also suggested [105,106] (Table 1). The mechanisms involved the activation of tumor-associated macrophages [85,86,87], suppressor myeloid cells [90,91] and the inhibition of NK cell activity [97,98,99]. By expressing PDL1 [92,93], the transforming growth factor (TGF) beta [94], the tumor necrosis factor–related apoptosis-inducing ligand (TRAIL) and the Fas ligand [95,96], cancer EVs exhibit an immunosuppressive activity on T cells. Moreover, cancer EVs inhibit the maturation of dendritic cells through a mechanism involving the expression of HLA-G [88] and specific miRNAs, such as miR-203 and miR-212-3p [89]. Despite tumor EVs have been mainly implicated in the tumor immune escape, they can also be exploited to cross-present tumor antigens to the antigen presenting cells eliciting an antigen-specific cytotoxic lymphocyte anti-tumoral response [100,101]. However, clinical trials based on this assumption have provided conflicting results [107,108,109].

At present, most of the studies looking for exRNAs as cancer biomarkers have been performed on whole blood, urine and cerebrospinal fluids. Recently, several studies have explored the detection of exRNAs associated with the EVs. Despite the fact that the quantitative and stoichiometric analyses revealed that many miRNAs are present in less than one single copy per single exosome [110], several studies indicate a potential utility as cancer biomarkers [102,103]. For instance, in hepatocellular carcinoma, the expression by serum EVs of miR-18a, miR-221and miR-224 has been suggested as potential diagnostic biomarkers [104]. Fabbri et al. demonstrated that EV-associated miR-21 and miR-29a bound to a Toll-like receptor family favoring an inflammatory pro-metastatic response in lung [85]. On the other hand, the expression of miR-21 in serum EVs in patients with breast cancer correlates with a favorable outcome [111]. By comparing the miRNA signature of ovarian cancer EVs with that of EVs from normal subjects, Taylor and colleagues suggested a potential utility to screening asymptomatic patients [102]. A significant similarity of EV-associated miRNAs was observed with tumor-derived miRNAs in lung adenocarcinoma [103]. Moreover, the miRNA patterns of patients were clearly distinct from those of normal controls, suggesting that circulating EV-associated miRNAs might be useful as a non-invasive screening test [103].

## 4. Salivary EVs as Biomarkers

EVs are particularly enriched in saliva, which in respect to blood does not undergo coagulation. This is an important issue because many studies have been performed on serum. Coagulation induces a consistent release of EVs from platelets, thus modifying the composition of circulating EVs [112]. Salivary EVs should derive in part from salivary glands and in part from circulation: indeed, about a 30% similarity of salivary and plasma proteome has been described by a few studies [113,114,115]. In particular, using liquid chromatography and mass spectrometry, 19,474 unique peptides have been isolated from whole saliva in a multicenter study [113]. Protein annotation was assessed by matching the identified peptides with a recently published dataset of the human plasma proteome [116], and 1939 different proteins were identified as commonly expressed in blood and saliva. However, a puzzling aspect is the expression of neuronal markers in salivary EVs with significant changes in the miRNA pattern and in the proteomic profile after a head concussion [117] and in neurological diseases [118,119]. Moreover, the EV composition may be affected by the presence in saliva of viruses, including the human papillomavirus (HPV) [120,121,122,123,124] and the neurotropic human herpesviruses (e.g., HHV-6), which are detectable in the saliva of infected subjects [125].

A critical aspect in the use of salivary EVs as biomarkers is the purification technique that is used (Table 3). In fact, results may vary depending on the purified subpopulations and the presence of contaminants, such as bacterial flora. Therefore, accurate mouth washing, careful standardization on saliva collection and sample filtration are recommended to abate the bacterial load.

Differential ultracentrifugation or density gradient ultracentrifugation are considered the gold standard for the purification of EV subpopulations. These techniques have been further implemented with the combined use of the immune-affinity capture of exosomes [34]. To improve the separation of vesicles from non-vesicular components a floating technique has been proposed, based on gradient fractioning centrifugation, with samples applied to the bottom of tubes [135]. However, the standardization of these techniques may be difficult, as the results are influenced not only by the centrifugal radius of the rotor and g force type, but also by the viscosity of the starting solution. In addition, due to mechanical damage, membrane debris are generated, as seen by electron microscopy. Moreover, the difficult detection of proteins and RNAs has been described [136,137,138,139]. To avoid shear stress due to ultracentrifugation, size exclusion chromatography has been employed with the aim to separate small vesicles from protein contaminants [140,141,142]. Immuno-affinity purification allows for the recovery of sub fractions of EVs based on the expression of surface markers [139,143,144,145], and several kits are commercially available. Microfiltration has also been used with membranes with appropriate pore sizes to remove cell debris and apoptotic bodies [143]. However, this technique is limited by EV adhesion to membranes and pore clogging. In addition, to isolate small biological samples, all these techniques may have a low efficient recovery of EVs. Another approach for isolating EVs from biological liquids is based on polymeric precipitation [146,147,148,149,150,151]. This approach allows for a rapid precipitation of EVs, but it is limited by the co-precipitation of proteins of a non-vesicular origin such as lipoproteins [136,152,153]. Recently, a new technique based on electric field-induced release and measurement has been successfully applied to liquid biopsy in saliva [154]. Using this technique, the mutation of epidermal growth factor receptor (EGFR) in patients with lung cancers was detected and matched with biopsy genotyping [154,155]. Moreover, the electric field-induced release has been combined with the magnetic beads immune-capturing of exosomes [156,157], resulting in a highly sensitive and specific method of exRNA extraction and analysis. Compared to polymeric precipitation and differential centrifugation, this approach is less time consuming, requires smaller sample volumes and does not involve sample lysis that may reduce exRNA yield. However, for each EV extraction, the capture probe that is attached to the magnetic beads allows for the isolation of only those EVs containing the exosome-specific surface marker used for capturing EVs [156]. In fact, EVs are a heterogeneous population of vesicles, and individual EV analyses show that not all EVs co-express the same tetraspanin. Therefore, this technique may not include the whole pattern of EV-associated exRNA.

By quantitative nano-structural and single molecule force spectroscopy, Sharma et al. [126] performed a bio-molecular analysis of exosomes present in the saliva from patients with oral cancer. They demonstrated that exosomes were augmented in number and size, displayed a dissimilar morphology and showed an increased expression of CD63. Similarly, Zlotogorski-Hurvitz et al. [127] described a bigger salivary exosome concentration and size in patients with oral cancers in comparison with healthy subjects, a higher expression of CD63 and a decreased expression of CD9 and CD81. Few other studies performed a proteomic analysis of salivary exosomes in search of potential biomarkers of oral [128] and lung carcinomas [130]. A higher expression of the CD63 molecule was observed in EVs from the saliva of patients with oral cancers in respect to normal subjects [126]. Sun et al. performed a comparative proteomic analysis of salivary EVs in normal subjects and lung cancer patients [131]. In this study, several proteins were found to be dysregulated, and four of them were present in both salivary microvesicles and exosomes, suggesting their potential use for the detection of lung cancer.

It has been reported that in saliva, the bulk of miRNAs is packaged in exosomes [13]. In fact, miRNAs are easily detectable in EVs present in saliva [158,159]. Several studies focused on the possibility of exRNA isolation from saliva and oral samples [160,161,162] and in particular on salivary EV associated miRNAs in patients with oral cancer [160,161,162,163,164].

Langevin et al. performed a comprehensive miRNA sequence analysis of EVs derived from the saliva of patients with head and neck carcinomas and identified a distinct pattern of secretion and, in particular, miRNAs secreted only by cancer cells [132]. Some miRNAs, such as miR-486-5p, miR-486-3p and miR-10b-5p, were specifically overexpressed in the EVs of a subset of head and neck carcinomas. Machida and colleagues showed that miR-1246 and miR-4644 present in salivary EVs are potential biomarkers of cancers of the pancreato-biliary tract [133]. Taken together, these analyses may provide the bases for the development of new tumor biomarkers (Table 3).

A transcriptomic signature specific for pancreatic [165] and ovarian cancers [166] and proteomic signature modifications in lung cancer [167] have been described in whole saliva. Zhang et al. [165] demonstrated that the combination of KRAS, metyl CpG binding domain protein 3 like 2 (MBD3L2), acrosomal vesicle 1 (ACRV1), and dolichyl-phosphate mannosyltransferase subunit 1 (DPM1) mRNAs in saliva may differentiate patients with pancreatic carcinomas from patients with chronic pancreatitis and healthy subjects with a high sensitivity and specificity. Moreover, a transcriptomic analysis of salivary EVs by next generation sequencing showed the presence of many coding and non-coding RNAs, such as mRNAs for several proteins, miRNAs, snoRNAs, piRNAs, and lncRNAs [48,168]. Palanisamy et al. [169] found, in exosomes isolated from saliva, 509 mRNA transcripts, which once incorporated in keratinocytes were able to modify the protein expression in these cells. Moreover, exosomes from adenocarcinoma of the pancreatic ducts were able to modify the biology of exosomes derived from the salivary gland and induce changes in the salivary biomarker profiles [134]. Similarly, they showed an interaction between exosomes derived from the human metastatic mammary gland epithelial adenocarcinoma cell line MDA-MB-231 cells and exosomes derived from the human submandibular gland (HSG) cells. This interaction induced an activation of the HSG cell transcriptional machinery with an increase of total cellular RNA and transcriptomic and proteomic changes [170]. Salivary EVs derived from patients with pancreatic carcinoma were shown to inhibit NK cell activation, thus favoring tumor immune escape [171].

**Figure 1:**
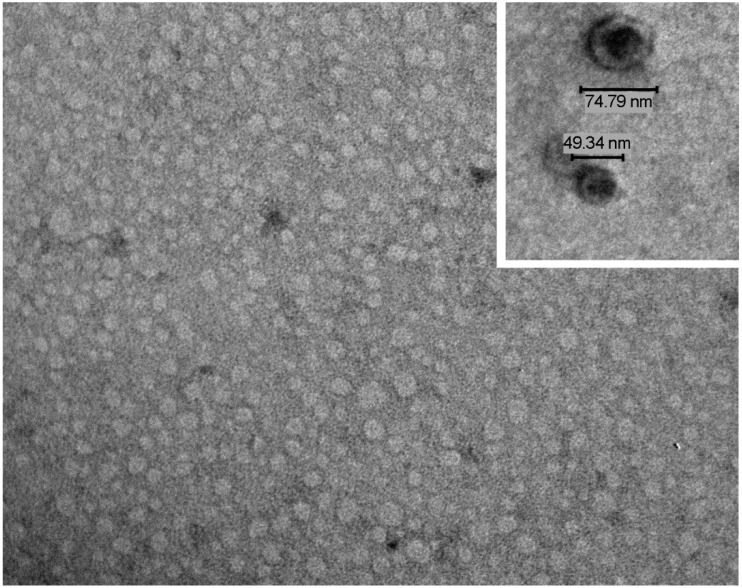
Salivary EVs characterization. A representative transmission electron microscopy image of EVs isolated by a charge-based precipitation method, showing a carpet of vesicles in the nano-range. In the inset, the bars indicate the size of the extracellular vesicles (EVs). The preparation was stained with NanoVan (JEOL Jem-1010 electron microscope, original magnification ×75,000; inset ×150,000).

We analyzed the zeta potential of salivary EVs and, based on their negative charge, we developed a charge-based precipitation protocol. This technique allows for the efficient recovery of exRNA from a salivary EV population with a very homogeneous size and shape [159] (Figure 1). In a recent work [129], we used the charge-based precipitation method to isolate EVs from the saliva of patients with oral squamous cell carcinoma (OSCC) to investigate the presence of exRNAs suitable as biomarkers. Our aim was to assess whether this quick, simple and efficient technique could be useful for detecting exRNA in the salivary EVs of patients with OSCC. The diagnosis of OSCC is based on oral examination and histological analysis. However, the identification of salivary biomarkers may have potential prognostic and therapeutic values. To exclude misleading results due to a different exposition to risk factors, at the time of patients’ recruitment, subjects included in our study were checked for their habits regarding smoking and alcohol consumption. In fact, smoke and alcohol consumption has been described as potentially affecting the composition of EV-associated exRNA [172,173]. Therefore, patients and controls were matched to obtain a similar distribution of risk factors among the two groups to reduce this bias. Moreover, 5 out 21 patients were positive to the Human Papilloma Virus (HPV). To avoid the detection of exRNA of viral origin, we screened EVs for the presence of about 800 miRNAs with human-specific primers. Although HPV infection may alter the EV release and cargo, we did not observe any significant change in the size and concentration of EVs from HPV-positive patients compared to negative patients. A differential expression of the EV miRNA signature in OSCC cells infected or not by HPV has been previously shown [124]. In this study, the authors observed that HPV infected cells released EVs enriched with 14 miRNAs, whereas non-infected cells overexpressed 19 miRNAs. The cohorts of patients we studied were too small to draw any conclusions. However, we did not observe the differential expression of miRNAs, which has been previously described for EVs released by in vitro infected OSCC cells.

By comparing the miRNA expression of cancer patients and matched controls, we observed an up-regulation of miR-412-3p, miR-512-3p, miR-27a-3p and miR-494-3p in patients with oral squamous cell carcinoma. MiR-512-3p and miR-412-3p were also potentially sensitive and specific biomarkers, as indicated by the high AUC values (0.847 and 0.871 respectively, with *p* values < 0.02) and a maximum Youden’s Index. Interestingly, we also observed an exclusive expression of miR-302b-3p and miR-517b-3p in cancer EVs. Moreover, we performed a bio-informatic analysis to better understand whether the tumor-enriched miRNAs could be functionally related to the tumor. We observed that eight tumor-related pathways were potentially targeted by these miRNAs. In particular, miR-512-3p and miR-27a-3p may target 7 and 20 genes, respectively, of the ErbB signaling pathway, which is known to promote cell proliferation and survival in cancer [174] and is activated in oral carcinomas [175,176,177]. MiR-512-3p, miR-27a-3p, and miR-302b-3p could potentially target proteoglycan genes and CD44 involved in c-Fos-mediated cell invasion and migration [178], ERK1/2 phosphorylation [179] and the phenotype of oral cancer stem cells [180]. Moreover, miR-512-3p, miR-412-3p, miR-27a-3p, and miR-302b-3p reduced the expression of TGFβR2, frequently reduced in cancer and stroma cells in patients with oral squamous carcinomas [181]. Increased levels of the oncogenic miR-27a-3p has also been detected in EVs obtained from the plasma of OSCC patients [182]. In this study, a comparable miRNA signature was observed between plasma EVs and EVs released by OSCC cells in vitro.

Recent studies have shown that EVs also contain lncRNAs [183]. The expression of lncRNAs has not been investigated in salivary EVs. However, salivary lncRNAs may represent a potential marker for OSSC [184]. In fact, a subset of lncRNAs was correlated with high metastatic OSCC. In particular, the lncRNA HOTAIR was found to be highly expressed in the saliva of patients with lymph node metastasis. Therefore, besides miRNAs, the search for lncRNAs in salivary EVs could be a valuable diagnostic and prognostic tool for OSCC.

## 5. Conclusions

Taken together, these studies suggest that EVs derived from cancer cells may modulate the function and may induce epigenetic changes in neighboring or distant cells. These biological effects are related to the delivery of transcripts that are specific of the originator cells. Several studies have shown a prominent role of exRNAs associated with vesicles. Since EVs may retain the molecular signature of the cell of origin, it has been suggested that they are a potential diagnostic exploitation. The salivary EV composition may reflect the presence of local or systemic diseases and has been investigated as a potential biomarker for both oral and non-oral cancers. Changes in the molecular composition of the EVs of non-oral cancers may either depend on their derivation from blood (since salivary glands are vascularized) or be the consequence of phenotypic changes occurring in gland cells (as the results of the stimulation by circulating cancer EVs). However, so far, available studies are relatively few and include a low number of patients. Further studies are necessary to optimize the protocol of EV isolation from saliva in order to obtain reproducible results. Moreover, the use of the EV content as a biomarker should take into account that this may be influenced by a number of cancer-associated risk factors, such as viral infections, smoking, alcohol abuse, as well as a number of non-cancer-associated factors related to concomitant diseases. However, these limitations in the use of EVs as biomarkers are not restricted to saliva, but may influence EVs derived from any biological fluid. Since saliva is an easily obtainable non-invasive bio-fluid particularly enriched in EVs, it may represent a new approach for cancer biomarker discovery. However, to define whether salivary EVs have a real clinical diagnostic and prognostic potential would require comparative studies between EVs derived from tumor cells, blood and saliva, which are not at present available.

## Figures and Tables

**Table 1:** The role of EVs in chemo-resistance and immune-modulation. Several proteins and exRNAs have been described to be involved in tumor chemo-resistance and immune-modulation.

Biological Effect	Mechanism of Action	Cell Source	Target	References
Resistance to chemotherapy	Transfer of MDR-1/P-gp	Docetaxel-resistant prostate cancer	Docetaxel-sensitive prostate cancer	[59]
	Transfer of miR-100, miR-222, miR-30a and miR-17	Adriamycin and docetaxel-resistant breast cancer	Adriamycin and docetaxel-sensitive breast cancer	[60]
	Transfer of miR-21	Platinum-resistant ovarian cancer	Platinum-sensitive ovarian cancer	[61]
	Transfer of miR-100-5p, miR-21 and miR-133b	Cisplatin-resistant lung cancer	Cisplatin-sensitive lung cancer	[62,63]
	Transfer of miR-21, which downregulates APAF1	Stroma	Ovarian cancer	[74]
	Transfer of miR-146a with Snail mRNA	Cancer-Associated Fibroblasts	Pancreatic cancer	[73]
	Activation of the antiviral/ NOTCH3 signaling pathway	Stroma	Breast cancer	[72]
Tumor immune-escape	Release of pro-inflammatory cytokines by macrophages, possibly mediated by miR-21 and miR-29a	Breast and lung cancer, melanoma	Tumor cells, fibroblasts, endothelial cells, and immune cells	[85,86,87]
	Inhibition of dendritic cell maturation and functions, by delivering specific miRNAs (e.g., miR-203, miR-212-3p)	Renal carcinoma, pancreatic cancer, melanoma	Dendritic and T cells	[87,88,89]
	MDSCs activation, which leads to TGF-β-mediated suppression of T cell activity	Melanoma and colorectal carcinoma	CD14^+^ monocytes	[90,91]
	Suppression of the T-cell activity mediated by PDL-1, TGF-β, Fas ligand and TRAIL	Melanoma, colorectal, gastric and prostate cancer, head and neck squamous cell carcinoma	CD8^+^T cells	[92,93,94,95,96]
	Inhibition of NK cell cytotoxic activity, possibly mediated by MIC A ligand of NKG2D receptor	Mammary carcinoma, melanoma, cervical, head and neck, liver cancer	NK cells	[97,98,99]
Enhancement of immune response	Activation of a tumor antigen-specific immune response in humans	Melanoma and non-small cell lung cancer patients-derived dendritic cells	systemic administration	[100,101]

EVs: extracellular vesicles, MDR: multidrug resistance protein, APAF1: apoptotic protease-activating factor 1, MDSCs: myeloid-derived suppressor cells, TGF-β: trasforming growth factor-β, PDL-1: programmed death-ligand 1, TRAIL: tumor necrosis factor-related apoptosis-inducing ligand, NK: natural killer, MIC: MHC class I–related chain, NKG2D: NKG2-D type II integral membrane protein.

**Table 2:** Role of EVs as biomarkers of tumor progression. Cancer-derived EV content has been proposed as a tumor biomarker and has been related to several processes involved in tumor aggressiveness.

Biological Effect	Mechanism of Action	Cell Source	Target	References
Tumor biomarkers	Transfer of miR-21, miR-141, miR-200a, miR-200b, miR-200c, miR-203, miR-205 and miR-214	Ovarian cancer	Serum	[102]
	Transfer of miR-17-3p, miR-21, miR-29a, miR-106a, miR-146 miR-155, miR-191, miR-192, miR-203, miR-205, miR-210, miR-212 and miR-214	Lung cancer	Serum	[85,103]
	Transfer of miR-18a, miR-221 and miR-224	Hepatocellular carcinoma	Serum	[104]
Pro-angiogenic effect	Transfer of proangiogenic miRNAs, mostly regulated by HIF-1α (miR-155-5p, miR-210 and miR-494)	Melanoma, hepatocellular, lung and renal adenocarcinoma	CAFs and endothelial cells	[75,77,78,79,81]
Decrease cell-to-cell adhesion	Reduction of E-cadherin, let-7i and β-catenin expression, and increase of Snail1-2, Twist1-2, Sip1, vimentin, ZEB2 and N-cadherin expression, activation of MAPK pathway	Breast and bladder cancer, melanoma	Mammary and urothelial cells epithelial cells, primary melanocytes	[67,68,69]
Increase in cell migration/invasion	Lipids and proteins (e.g., CD81)-dependent stimulation of the cancer cell motility via Wnt signaling	Cancer Associated Fibroblasts	Melanoma, breast and prostate cancer	[70,71]
Development of premetastatic niche	Delivery of TYRP2, VLA4, HSP70, an HSP90 isoform and the MET oncoprotein	Melanoma	Bone marrow progenitor cell	[83]
	Exosomal expression of tumor-specific integrin patterns	Osteosarcoma, rhabdomyosarcoma, Wilms tumor, skin and uveal melanoma, breast, colorectal, pancreatic and gastric cancer	Brain, lung and liver epithelium	[82]
	Delivery of MIF	Pancreatic ductal adenocarcinoma	Kupffer cell	[80]
	Delivery of specific oncogenic miRNAs, e.g., miR-125b, miR-130b and miR-155, which induce a neoplastic reprogramming of recipient cells	Prostate, renal cancer	Adipose-derived stem cells, lung epithelium	[81,84]

HIF-1a: hypoxia inducible factor 1α, HSP90: heat shock protein 90, MET: hepatocyte growth factor receptor., TYRP2: tyrosinase-related protein-2, VLA4: very late antigen 4, HSP70: heat shock protein 70, MIF: macrophage migration inhibitory factor.

**Table 3:** Biomarkers detected in salivary EVs. Salivary EVs can be purified using different EV isolation techniques and can be exploited as biomarkers because they contain disease-related proteins and exRNA.

Disease	Isolation Method	EV Biomarkers	Type of Biomarker	References
Brain injury and neurological disorders	Differential ultracentrifugation	CDC2, CSNK1A1, and CTSD	mRNA	[117]
	XYCQ EV Enrichment KIT	α-synuclein	protein	[119]
Oral squamous cell carcinoma	Differential ultracentrifugation	CD63	protein	[126,127]
	Differential ultracentrifugation	PPIA	protein	[128]
	Charge-based precipitation	miR-412-3p, miR-512-3p, miR-27a-3p, miR-494-3p, miR-302b-3p, miR-517b-3p	miRNA	[129]
Lung cancer	Affinity chromatography column combined with filter system (ACCF)	Annexin A1, A2, A3, A5, A6, A11; NPRL2; CEACAM1; MUC1; PROM1; HIST1H4A; TNFAIP3	protein	[130]
	Affinity chromatography column combined with filter system (ACCF)	BPIFA1, CRNN, MUC5B, IQGAP	protein	[131]
Head and neck carcinoma	Differential ultracentrifugation	miR-486-5p, miR-486-3p, miR-10b-5p, miR-122	miRNA	[132]
Pancreatic cancer	Total Exosome Isolation Reagent (Invitrogen)	miR-1246, miR-4644	miRNA	[133]
	Differential ultracentrifugation	Apbb1ip, Aspn, BCO31781, Daf2, Foxp1, Gng2, Incenp	mRNA	[134]

CDC2: Cyclin-dependent kinase A-1, CSNK1A1: Casein Kinase 1 Alpha 1, CTSD: Cathepsin D, PPIA: Peptidyl-prolyl cis-trans isomerase A, NPRL2: GATOR complex protein NPRL2, CEACAM1: Carcinoembryonic antigen-related cell adhesion molecule 1, MUC1: Mucin 1, PROM1: Prominin 1, HIST1H4A: Histone H4, TNFAIP3: Tumor necrosis factor alpha-induced protein 3, BPIFA1: BPI fold-containing family A member 1, CRNN: Cornulin, MUC5B: Mucin 5b, IQGAP: Ras GTPase-activating-like protein IQGAP1.
